# Supplementary material for: Inhibition of 37/67kDa Laminin-1 Receptor Restores APP Maturation and Reduces Amyloid-β in Human Skin Fibroblasts from Familial Alzheimer’s Disease
Source: J Pers Med. 2020 Nov 16;10(4):232. doi: 10.3390/jpm10040232 (PMC7712490; doi:10.3390/jpm10040232)
Supplement: Supplementary file 1 [file jpm-10-00232-s001.pdf]

Supplementary material

Inhibition of 37/67kDa laminin-1 receptor restores APP maturation and reduces Amyloid-β in human skin fibroblasts from familial Alzheimer's disease

Antaripa Bhattacharya[1,†], Antonella Izzo[1,†], Nunzia Mollo[†1], Filomena Napolitano[2], Adriana Limone[1], Francesca Margheri[3], Alessandra Mocali[3], Giuseppina Minopoli[1], Alessandra Lo Bianco[4], Federica Di Maggio[5], Valeria D'Argenio[5,6], Nunzia Montuori[2], Antonio Lavecchia[4], Daniela Sarnataro[1,5*]

Table S1. Full list of the primer pairs used to amplify APP, PSEN1 and PSEN2 genes.

Gene	Transcript	Exon	Forward 5'->3'	Reverse 5'->3'	Product Lenght (bp)
APP	ENST00000357903.7	1	TTTCCTCGGCAGCGGTAG	GACTAAGTCGGGGTCTGGG	500
		2	GAAGACCGGGCTGATTCCTAATT	ACTGTAGGGTTAAAATACTGATGCA	400
		3	TCCGGATTCCAGATGCTCAA	TGCAAGAGTCCAAAACACAGT	481
		4	GCTTAAGGAGGCCAGACGTA	GGGGTACACGGGAACTCTAT	496
		5	TGGGTCTGCATGTTGATTATTTT	TGGGCAGAGACCTTTTCAGT	393
		6	CCACTGAGCTGGGATTTATACT	GCCAAGCAGCATATACAAGGT	587
		7	GGTGGCCAGTTAAATTCCTCA	TGAACATACTGCGGAGACTCT	494
		8	CTGCACAGTGTCTCATGGTG	CAGCATGTTCACGGAGGATG	489
		9	AGTGGGAGGTCAAATATTCTTCA	TGGGAGCAAATATAAGGCAGGA	422
		10	AGTGCAAGAAAGTGAAGTAAGCT	GACCTTCAAGATGGAATGGACA	499
		11	GCAGCCGATCTAAGTAATTACCC	TGCCAAACCACATATTGCTCT	496
		12	TCAAGTCTTAAACCTGAGCTCA	GGCGACATTCCTCCAGTCTT	581
		13	ATGTCATGGCTCTAAACGCG	GGCCCTTTACCTGCAAATGT	688
		14	AACTAAGGAGCAGGACTGGC	TCACTCGGAACTTGGGAAATG	476
		15	AGCATGTATTTAAAGGCAGCAGA	CCTTAATTTGATTTCTAGCACAGGA	443
		16	CCAGTTGGGCAGAGAATATACT	CCTTGAGCAGAATATTCACGGT	495
		17	GTGTAACTGGTGGGCAAAGG	GTGGATTAATTCAAGTTCAGGCA	457
PSEN1	ENST00000324501.9	2-3	TCAGTTAGTTGAAAGTCGTGACA	AAGGCTTCAACTGAGGTGGT	671
		4	AAAGAGAGGACCTGAATGCC	AGCATAACTTCAGCCCTATCCA	694
		5	TAGATGGAGCCAGTGTCTGC	TCTGAGCCTGGCATTACACA	598
		6	AAAGTTGTTTTAAAGAGAGATGTGG	ATCTTCAAAAAGGCTTAGAATTAACTG	700
		7	GAAGACCACCTGATCTCCGG	GCCCAGCCGAAATCTTCAAA	688
		8	AGACTTGTTCCTATACCCCAGT	GGAATCCTTGAGCTTGCCCT	679
		9	TCTTCTCTTACCTGCTAAAACCA	TGCTTTGACTGTATTGTTGGGT	465
		10	TAAAGGAGAAAATAGCTGTTGGATAAA	TCTCAATTCATTTTATTCTCAAAAAGG	434
		11	CTCATTCATTGTGGGGTTGAGT	TTCTAATGTGTGGCCAGGGT	397
		12	TGTGTGCATAATGAACCCTATGA	GTCCACTGCGATGAAGTATGT	585
PSEN2	ENST00000366783.7	4	CCTGACTTTCGTGGCTATGC	AAACCCTGCAGATGGAAGGT	594
		5	TGCCCTAGTAGCTCATAGACTG	CGGTTCTTCATCCCTGCTCT	492
		6	GAACTGCTCATGGGGATGGT	TCTAAAGGCGGCTGTTTCAC	389

7	GGAACTCCTTGGACTTCTGTG	GGTTCCCAAAACGGCTGG	386
8	GCATCCCCACCTTGTTTTAGA	TGCGTTTTATCTAGACCACCAG	600
9	CCCTACTCTGGCTCACACC	GATGCCTGCTTCCTGTCCTA	480
10	TGTGAGGTCTTGGCTCTGAT	ATCTTCAAGCCGCATGTGTC	500
11	CCTCTGCCAGCCTCTGTT	GGACAACTACATGGGGCCT	493
12	CATGTCCCCAGTCCACATCT	CCTGCTCTTGTCCCTCAGAG	399
13	GCCCTTAACACCTCAAGAGC	GAAACAGCTGGCACCAAAGA	499

Bp: base pair.

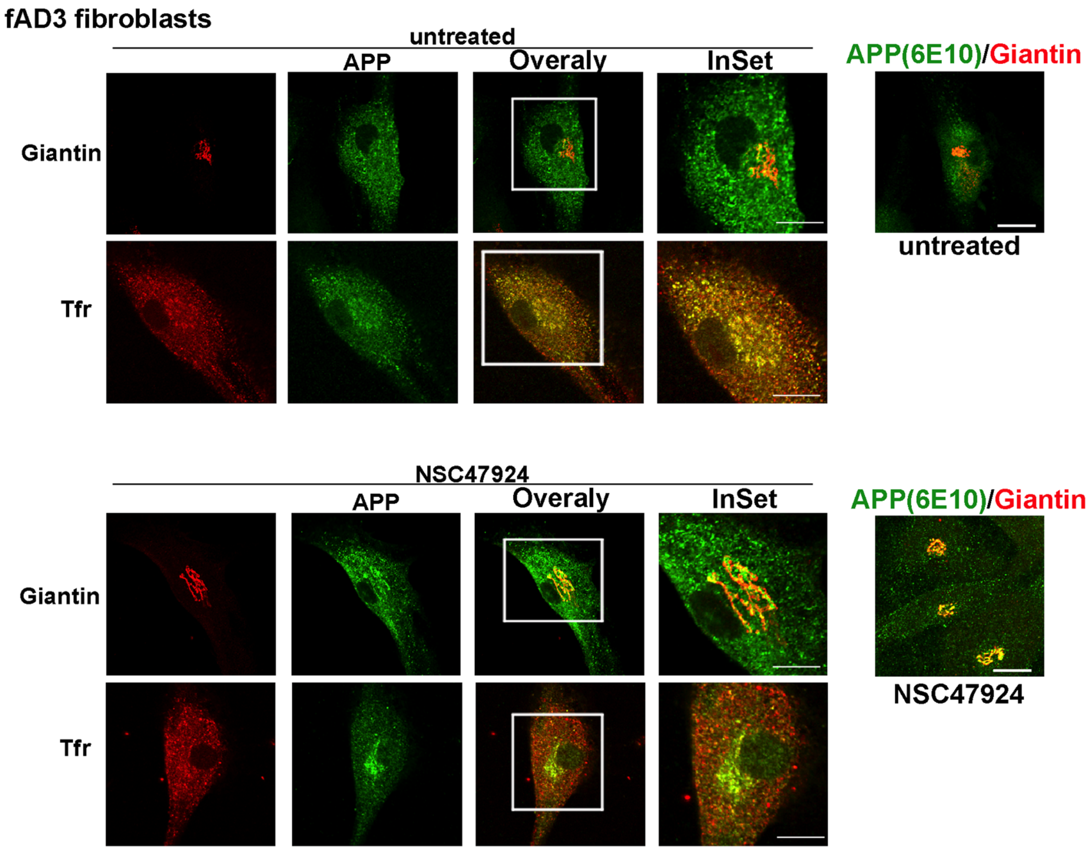

Figure S1. APP relocates in Golgi apparatus and the amyloid beta does not accumulate inside fAD cells, after NSC47924 treatment. FAD3 cells were grown and processed for immunofluorescence as in Figure 2. Note the absence of APP in the Golgi apparatus and the presence in recycling endosomes in untreated fAD fibroblasts. Intracellular APP localization is reversed by NSC47924 incubation. Panels on the right show cells double labelled with anti-APP 6E10 antibody (directed against 1-16 epitope of Aβ, green), and with anti-Giantin antibody (right panels, red), under untreated and NSC47924 treated conditions. Scale bars, 10 µm.

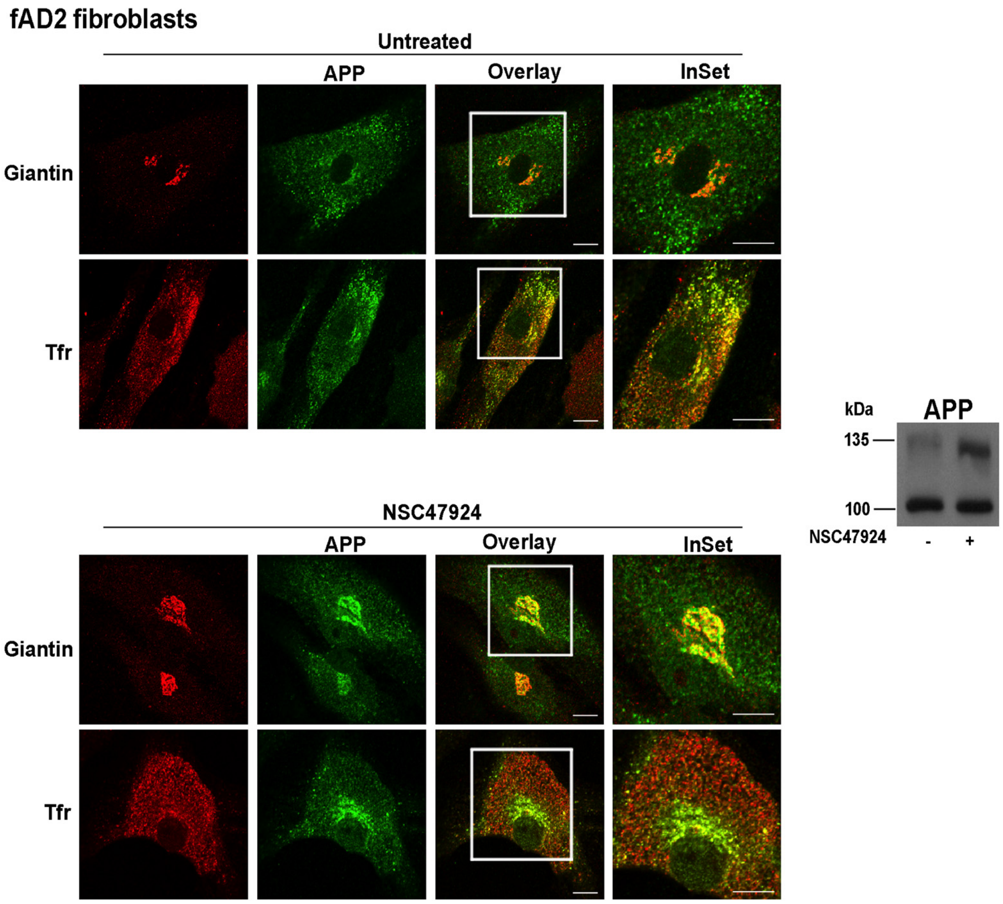

Figure S2. APP is mainly localized in Tfr-positive recycling endosomes in fAD2 fibroblasts and relocates in the Golgi after inhibitor treatment. FAD2 cells were grown and processed for immunofluorescence as in Figure S1. Note the absence of APP in the Golgi apparatus and the presence in recycling endosomes in untreated fAD fibroblasts. Intracellular APP localization is reversed by NSC47924 incubation. Scale bars, 10 µm. Note the increment of mature APP isoform (135kDa) in NSC47924 treated cells (western blot panel on the right).

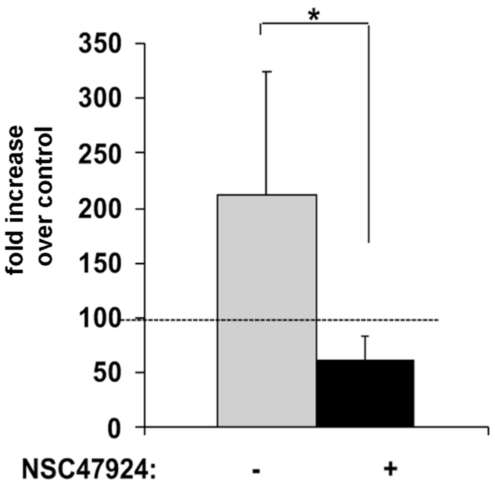

Figure S3. Aβ levels were decreased by NSC47924 compound in fAD cell culture media. Conditioned medium from fAD fibroblasts were incubated with anti-Aβ monoclonal antibody (4G8) or non-immune control immunoglobulins IgG. Bound anti-Aβ antibody was revealed by OPD staining; the absorbance at 490 nm was measured. Anti-Aβ antibody binding to BSA-coated wells was subtracted to obtain specific binding. Results are expressed as percent increase of anti-Aβ antibody absorbance value over non-immune control IgG, using as 100% the expression value in control conditions. Values represent the mean ± SEM of three experiments carried out in triplicate; (*P <0.05).
